# Disproportionate atherosclerotic burden in the left anterior descending coronary artery in participants without standard modifiable cardiovascular risk factors: The multi-ethnic study of atherosclerosis (MESA)

**DOI:** 10.1016/j.jcct.2026.01.004

**Published:** 2026-01-31

**Authors:** Michael P. Gray, Kristy P. Robledo, Anthony C. Keech, Stephen T. Vernon, Matthew J. Budoff, Gemma A. Figtree

**Affiliations:** a Faculty of Medicine & Health, University of Sydney, Sydney, NSW, Australia; b NHMRC Clinical Trials Centre, The University of Sydney, Sydney, NSW, Australia; c Department of Cardiology, Royal Prince Alfred Hospital, Sydney, NSW, Australia; d Department of Cardiology, Royal North Shore Hospital, Sydney, NSW, Australia; e Harbor-UCLA Medical Center, Lundquist Institute, Torrance, Calif., USA

**Keywords:** Atherosclerotic cardiovascular disease, Coronary artery calcification, Coronary heart disease, Standard modifiable cardiovascular risk factor (SMuRF)

## Abstract

**Background:**

Standard modifiable cardiovascular risk factors (SMuRF) have been identified for coronary artery disease (CAD) and targeted in primary prevention efforts, resulting in significantly improved outcomes. However, an increasing proportion of individuals present with ST elevation myocardial infarction (STEMI) in the absence of conventionally elevated levels of modifiable risk factors (“SMuRFless”), with significantly worse short-term outcomes. Culprit lesions of the left anterior descending artery (LAD) are more frequently implicated in SMuRFless STEMI patients than those with at least one risk factor. Differences in segmental-level coronary disease burden outside the acute setting in SMuRFless individuals are largely unknown.

**Objectives:**

To characterise vessel-level coronary calcification, comparing SMuRFless to SMuRF≥1 participants.

**Methods:**

Secondary analysis of the Multi-Ethnic Study of Atherosclerosis (MESA) was completed. Coronary artery calcium score (CACS) was measured at baseline and follow-up (median 6.3 years).

**Results:**

6792 participants were included, with 20.6 % classified as SMuRFless. Among participants with detectable coronary calcification at baseline, SMuRFless participants had a significantly higher proportion of their total coronary calcium localised to the LAD, compared to SMuRF≥1 participants (74.2 % vs. 58.9 %, p < 0.0001). This difference was most pronounced in individuals with higher baseline total CACS (CACS 100–400 Agatston units [AU] and CACS 401+), but attenuated after multivariable adjustment and was not statistically significant in best-subsets modelling. LAD CACS progressed in both groups during follow-up; however, annualised progression rates were similar between SMuRFless and SMuRF≥1 participants after adjustment for total coronary calcium progression and follow-up duration.

**Conclusion:**

SMuRFless MESA participants had a higher unadjusted proportion of coronary calcification in the LAD compared SMuRF≥1 participants; however, this associated attenuated after multivariable adjustment. Further research is warranted to better understand the development of atherosclerotic CAD in SMuRFless individuals, and biological relevance and potentially different susceptibility at the epicardial segment level.

## 1. Introduction

Atherosclerotic coronary artery disease (CAD) is the most common form of CVD and is responsible for approximately 46% of cardiovascular disease (CVD)-related mortality.^1^ Over the past fifty years, observational cohort studies have enabled the identification of fixed and modifiable risk factors associated with CVD-related mortality.^2^ Standard modifiable cardiovascular risk factors (SMuRF)—including hypertension, hypercholesterolaemia, diabetes mellitus, and tobacco smoking—have been the targets of both population- and individual patient-level primary prevention efforts, resulting in significant improvements in age-adjusted CAD-related mortality in both sexes in most of the world.^3,4^ However, patients continue to present acutely with ST elevation myocardial infarction (STEMI): the catastrophic clinical consequences of CAD. Efforts to control modifiable risk factors have resulted in epidemiologic changes in STEMI presentation characteristics. An increasing proportion of patients are presenting in the absence of SMuRFs (or “SMuRFless”).^5–7^ Despite a seemingly lower CVD risk profile, patients who have developed atherosclerotic STEMI in the absence of SMuRFs have been shown to have a significantly worse in-hospital^6,8^ and 30-day^9^ all-cause mortality compared to individuals with at least one SMuRF.

Since the initial observation of the surprisingly higher early mortality in SMuRFless STEMI patients, we have examined potential mechanisms and contributing factors. Initially thought to be a chance finding, the higher rate of the LAD culprit lesions in this population has since been extensively validated. In 3000 individuals presenting with first time STEMI to 42 Australian hospitals, 19 % were SMuRFless. These individuals had a 25 % higher rate of LAD territory acute lesion compared to those with at least one SMuRF (46 % vs. 37 %). This higher rate of LAD territory STEMI culprit lesions was confirmed in analyses of more than 62,000 patients from the SWEDEHEART study (41.5 % vs. 37.4 %),^9^ in 600 young patients aged 18–45 years (62.0 % vs. 47.2 %),^10^ nearly 24,000 STEMI patients in Singapore (57.3 % vs. 49.5 %),^7^ and 2000 participants in DANAMI-3 (50.2 % vs 41.7 %).^11^ Finally, a meta--analysis including nearly 1.3 million participants across 15 clinical studies reported a higher proportion of LAD culprit lesions in SMuRFless STEMI patients compared with their SMuRF positive counterparts (52.8 % vs. 47.2 %).^8^

The development and progression of coronary atherosclerosis is known to differ across the coronary tree, with certain territories appearing predisposed to early disease. Vascular territories exposed to lower shear stress, including wider vessel lumen and bifurcations, are believed to be more susceptible to atherosclerosis in the early phase of disease development.^12,13^ Non-contrast cardiac CT studies have demonstrated that coronary calcium, associated with more advanced plaque, preferentially localises to the proximal LAD.^14,15^ Coronary CT angiography (CCTA) studies have confirmed this pattern, identifying the proximal LAD as the most frequently affected coronary segment for atherosclerosis beyond calcified plaque.^16,17^ Prevalence of LAD atherosclerotic plaque has been shown to be significantly higher in individuals in the high 10-year ASCVD risk group compared to those with Low or Intermediate risk.^18^ The CONFIRM-based nested case-control ICONIC study of participants undergoing CCTA who went on to have an acute coronary syndrome (ACS) event, compared to matched controls, demonstrated a higher prevalence of LAD atherosclerotic lesions identified as a culprit lesion compared to non-culprit lesion.^19^ The presence of LAD atherosclerosis has potential prognostic significance, associated with poorer outcomes in individuals with chest pain compared to those with atherosclerosis in other coronary segments.^20^

To explore the predisposition to LAD territory disease in the SMuRFless population outside the acute coronary syndrome setting, we analysed coronary artery calcium score (CACS) data from the Multi-Ethnic Study of Atherosclerosis (MESA). Specifically, in this asymptomatic population, we compared LAD calcium between SMuRFless and SMuRF≥1 participants, assessed for an association between SMuRFless status and LAD calcification (after adjustment for baseline total CACS), and examined longitudinal LAD CAC progression by baseline SMuRF status in individuals with detectable coronary calcium.

## 2. Methodology

### 2.1. Study population

This study was completed as a secondary analysis of the MESA cohort. The protocol and primary study objectives of the MESA study have been previously described in detail.^21,22^ Broadly, the MESA study is a longitudinal, prospective, observational, cohort study seeking to improve the understanding of coronary atherosclerotic pathogenesis, specifically investigating the prevalence and impact of coronary calcium. 6814 participants free from previously diagnosed CVD were recruited by six U.S. clinical sites between 2000 and 2002.

### 2.2. Non-contrast cardiac CT

Coronary artery calcium scores (CACS) were measured at baseline for each participant. Participants recruited by half of clinical sites underwent electron-beam CT and half multi-detector row helical CT. Two CT scans were obtained for each participant at baseline with the results of these scans averaged. The amount of calcium was quantified utilising the Agatston method.^23^ Full CT analytical methods have been previously reported.^24^ Follow-up CT scans were performed at either study visit #2 (approximately 1.6 years after baseline visit) or study visit #3 (approximately 3.2 years after baseline), with participants randomly assigned to one of the two follow-up schedules.

To assess the distribution of coronary calcium across the major epicardial vessels, individual vessel calcium scores (left main, left anterior descending [LAD], left circumflex, and right coronary arteries) were expressed as a percentage of the total CACS: (segmental calcium score/total calcium score) x 100.

### 2.3. Baseline characteristics

An initial study exam was completed by all participants at enrolment. Information was collected on demographics, lifestyle, past medical history, relevant family history, and medications by questionnaire. Anthropometrics were collected by study staff. A fasted blood was collected for clinical pathology, including lipids, glucose, inflammatory markers, fibrinogen, and creatinine. Resting blood pressure was collected three times in a seated position, with the last two measures recorded for the study. Medications were verified by study staff during the baseline visit.

Participants were defined as “SMuRFless” if they did not meet any of the definitions of smoking, diabetes, hypertension, or hypercholesterolaemia below. “SMuRF≥1” was assigned for participants meeting one or more of these definitions.

A positive smoking history was defined as either: a) self-reported current smoker; or b) self-reported former smoker who quit less than one year ago. Diabetes was defined as: a) self-reported diabetes diagnosis; b) taking insulin or oral medication for diabetes; or b) fasting glucose ≥126 mg/dL (7 mmol/L). Hypertension was defined as: a) self-reported hypertension diagnosis; b) taking anti-hypertensive medications; c) systolic blood pressure (SBP) ≥ 140 mmHg; or d) diastolic blood pressure (DBP) ≥ 90 mmHg. Hypercholesterolaemia was defined as: a) self-reported diagnosis; b) taking lipid-lowering medications; c) LDL-C >135 mg/dL (3.5 mmol/L); or d) total cholesterol >212 mg/dL (5.5 mmol/L).^25^

Clinically relevant CACS groups were created from averaged baseline CT data as CACS 0, 1–99, 100–400, and 400+.^26^

### 2.4. Statistical methods

Baseline characteristics and coronary calcium (total Agatston score and percent of calcium in each epicardial vessel territory) were analysed, comparing SMuRFless to SMuRF≥1 participants. Continuous variables were assessed for normality using histograms. Normally distributed variables are reported as mean ± standard deviation (SD) and non-normal data presented as median (interquartile range, IQR). Categorical variables are reported as number (percentage). Between-group comparisons were completed with Student’s t-test for normally distributed continuous data, Mann-Whitney two-sample statistic for non-normal continuous data, and chi-squared test for categorical data.

Total and individual epicardial vessel calcium scores were transformed as Ln (Calcium Score + 1) due to the significant right-skew of the data and high prevalence of participants with a CACS of 0 Agatston units (AU). The relationship between LAD CACS and SMuRFless status at baseline status was first examined using a linear regression model adjusted for total CACS (bivariable models). Best-subset regression analyses were performed using baseline variables found to be *p*-value of <0.2 significant on univariate analysis, with age/sex forced into all models. Sensitivity analyses of coronary artery calcium volume by epicardial vessel was performed.

Differences in calcium distribution and progression were explored for all participants, as the difference in the first and the last available measurements. The progression of LAD calcium in participants with a positive CACS at baseline was explored as the relative change in log-transformed LAD CACS between the baseline and most recent CT (i.e., ln (LAD CAC_FU_ + 1) – ln (LAD CAC_BL_ + 1)) as previously described.^22^

A two-sided *p*-value <0.05 was considered statistically significant. No imputation was performed. Statistical analyses were performed with STATA/SE 17.0 (Stata Corporation LP, College Station, Texas, U.S.A.).

## 3. Results

### 3.1. Baseline characteristics

The initial MESA study population for analysis included 6792 participants with a baseline CT and data available for SMuRF classification, after excluding 22 (0.3 %) due to missing data regarding smoking status (Supplemental Fig. 1). 20.6 % (1397/6792) participants were classified as SMuRFless, having met no baseline criteria for hypertension, hypercholesterolaemia, diabetes, or smoking. Coronary artery disease (CACS>0) was identified at baseline in 50.0 % (3368) of participants. A positive CACS was found in 54.0 % (n = 2891) of SMuRF≥1 individuals and in 34.4 % (n = 477) of SMuRFless participants, indicating that coronary calcification was not uncommon even in the absence of conventionally elevated levels of modifiable risk factors. Baseline characteristics and coronary calcium data for the analysis population are presented in Table 1. Baseline characteristics for the full study population are presented in Supplemental Table 1.

We focussed our analysis for this study on individuals with evidence of CAD by CACS>0. SMuRFless participants with a positive CACS were younger, more frequently male and, by definition, had a lower cardiovascular risk factor burden compared to SMuRF≥1 participants as reflected in 10-year ASCVD risk score (Table 1). In addition, total CACS was significantly lower in these SMuRFless participants (57.6 vs. 102.2 AU, *p* < 0.0001).

### 3.2. Baseline association of LAD calcification and SMuRFless status in participants with a positive CACS

**Fig. 1.**
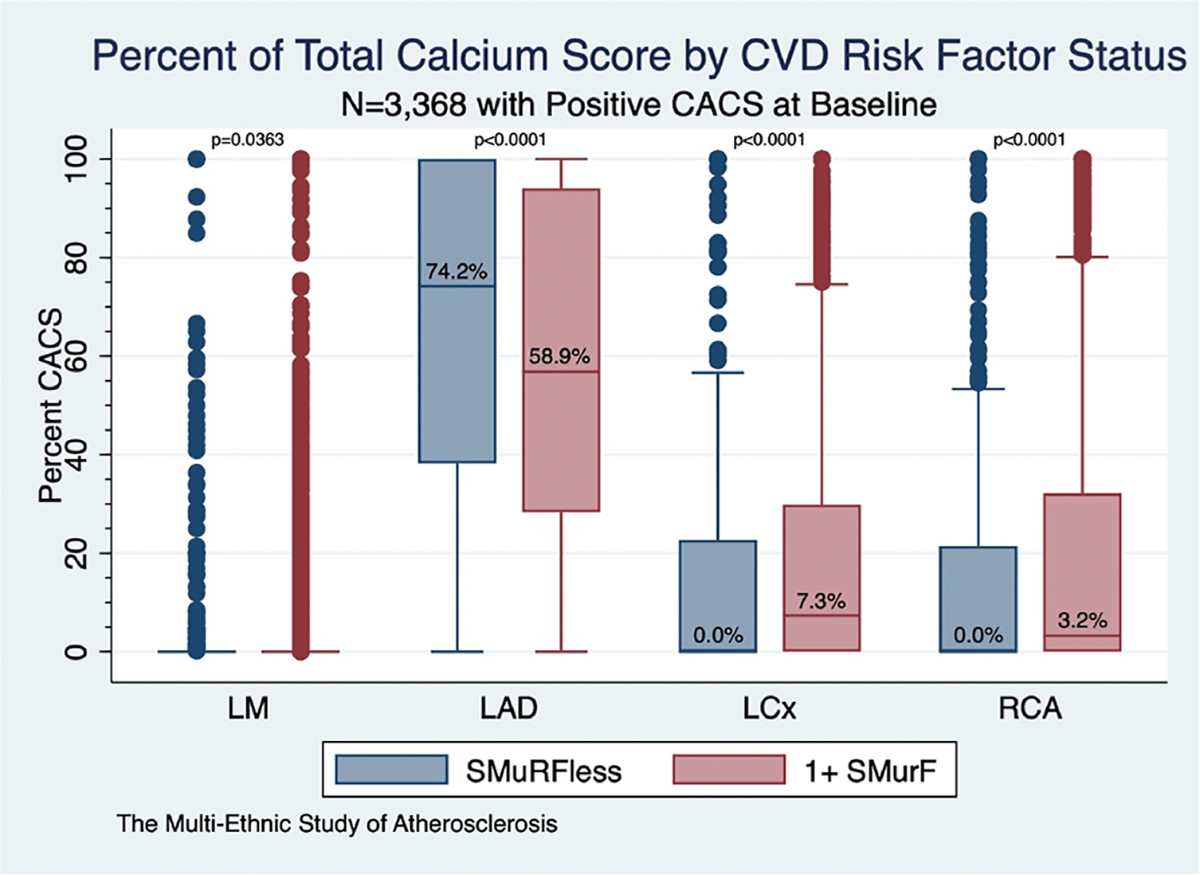
Proportion of total CACS in each epicardial vessel territory by SMuRFless status. Participants classified as SMuRFless had a significantly higher proportion of their total CACS in the LAD territory compared to participants with at least one SMuRF. This trend was reversed in the LCx and RCA territories when comparing these same groups. *P*-values based on Wilcoxon rank-sum test for non-normally distributed continuous variables. Boxplots depict distribution of vessel-specific percent contribution to total CACS. **CACS** = coronary artery calcium score; **LAD** = left anterior descending; **LCx** = left circumflex artery; **RCA** = right coronary artery; **SMuRF** = standard modifiable cardiovascular risk factor.

The proportion of total coronary calcification in each epicardial territory (left main, left anterior descending, left circumflex, and right coronary arteries) were considered. In participants with a positive CACS, the LAD accounted for the largest share of total coronary calcium (58.9 %, IQR 28.6–96.8 %). SMuRFless participants had a significantly greater proportion of LAD calcification compared to SMuRF≥1 participants (74.2 % versus 56.8 %, *p* < 0.0001) (Fig. 1).

**Fig. 2.**
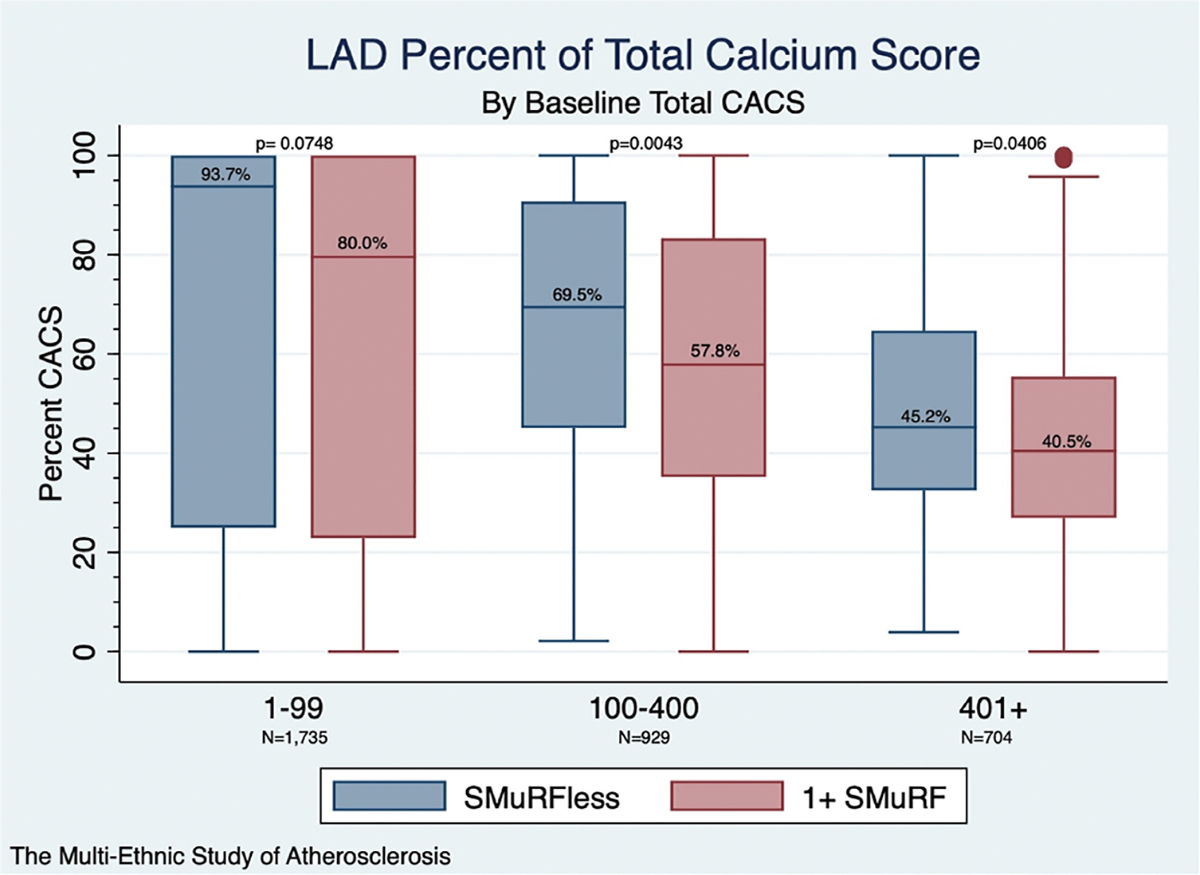
Proportion of total CACS in each epicardial vessel territory by SMuRFless status and clinically relevant CACS strata. Comparing SMuRFless and SMuRF≥1 participants within clinically significant total CACS strata demonstrated statistically similar proportions of LAD calcification in participants with CACS 1–99. However, CACS 100–400 and 400+ SMuRFless participants maintained significantly higher proportion of calcification in their LAD. **CACS** = coronary artery calcium score; **LAD** = left anterior descending; **SMuRF** = standard modifiable cardiovascular risk factor.

To evaluate whether this difference persisted across varying levels of total coronary calcium, we stratified participants by clinically relevant CACS groups. The proportion of total calcification in the LAD was statistically similar for SMuRFless and SMuRF≥1 participants in the CACS 1–99 subgroup (93.7 % versus 79.6 %, *p* = 0.07) (Fig. 2). However, when comparing SMuRFless to SMuRF≥1 participants within higher CACS strata, SMuRFless status was associated with a higher proportion of LAD CACS in the CACS 100–400 (69.5 % versus 57.8 %, *p* = 0.0043) and CACS 401+ (45.2 % versus 40.5 %, *p* = 0.0406) subgroups.

Given the relationship with total CACS burden on LAD distribution in all three groups with a positive CACS, we examined the association of SMuRFless status on total LAD CACS adjusting for total CACS at baseline (Table 2). Hispanic ethnicity and increasing BMI, fibrinogen, and CRP were associated with lower total LAD CACS, reflecting an association with calcification outside the LAD territory. A final model was created utilising best-subsets regression, after which only Hispanic ethnicity, increasing CRP, and total CACS were significantly associated with LAD CACS. The association between SMuRFless status and LAD CACS was attenuated in this model and no longer statistically significant after multivariable adjustment. Sensitivity analysis, modelling LAD coronary calcium volume in a similar strategy, demonstrated comparable results and suggested the identified relationships were not driven by calcium density (Supplementary Table 2).

To assess whether coronary calcification differences extended to other territories, we examined log-transformed LCx and RCA CACS in both bivariable and multivariable models. SMuRFless status was significantly associated with lower LCx CACS after controlling for total CACS, though this association was not significant after adjustment in the final model using Best-subsets. No differences were observed in RCA CACS (Supplementary Tables 3–4).

### 3.3. Progression of LAD calcification and SMuRFless status in participants with coronary calcium at baseline

Of the 3368 participants with baseline data and a positive CACS, 86.8 % (2925) completed at least one follow-up visit with a median follow-up time of 6.3 years (IQR 2.9–9.4) (Supplemental Table 5); follow-up time did not differ significantly between SMuRFless and SMuRF≥1 participants. Overall, 91.7 % of participants experienced CACS progression over the follow-up period, with similar rates of progression between SMuRFless and SMuRF≥1 participants.

Annualised CACS progression was calculated for all participants for total and segmental CACS (Supplemental Table 5). SMuRFless participants experienced a lower annualised CACS increase compared to SMuRF≥1 participants (12.7 vs. 23.1 AU/year, *p* < 0.0001), a trend that was similar for each of the epicardial vessel territories. LAD CAC annual progression was higher in those with a higher baseline CACS and did not significantly differ between SMuRFless and SMuRF≥1 participants (Table 3). Interaction terms for CACS strata and SMuRF status were not statistically significant for either sex, demonstrating the effect of SMuRF status appears consistent over CACS strata. The annualised change in LAD CAC was similar between SMuRFless and SMuRF≥1 participants when controlling for total CACS (Table 4). Further adjustment including statin initiation after baseline was non-significant and did not modify these findings.

## 4. Discussion

In this study, we evaluated coronary calcium distribution in individuals without modifiable CVD risk factors at baseline, with particular focus on significantly increased calcium burden in the LAD territory. Amongst MESA participants with a positive CACS, those classified as SMuRFless at baseline had a significantly higher proportion of coronary calcium localised to the LAD compared to SMuRF≥1 participants (74.2 % vs. 58.9 %, *p* < 0.0001). Across clinically significant CACS strata, the relative proportion of LAD calcification decreased with increasing total CACS burden; however, SMuRFless participants continued to demonstrate higher LAD involvement in the 100–400 AU and 400+ categories. This relationship persisted after adjustment for total CACS, but attenuated in multivariable modelling including Hispanic/Latino ethnicity and CRP losing significance, indicating part of the observed difference may be explained by other demographic and inflammatory factors. The persistence of CRP as a significant covariate in the final model highlights the potential role of systemic inflammation in development of atherosclerosis, including in individuals without risk factors.

SMuRFless participants in the MESA study with a positive baseline CACS had a similar rate of progression of their LAD CACS when controlling for baseline compared to SMuRF≥1 participants in univariable and multivariable analysis. This disparity between a higher proportion of total coronary calcification residing in the LAD in SMuRFless participants, seemingly driven by greater numbers of participants with higher total CACS at baseline and similar rates of annualised progression suggests a potential biological or time-dependent phenomenon to be explored.

The paper focuses specifically on the LAD due to its well-established clinical relevance and association with worse cardiovascular outcomes during an acute event. Prior studies have highlighted worse short-term outcomes amongst SMuRFless individuals presenting with STEMI, often with a higher proportion of LAD culprit lesions. The anatomical and hemodynamics of the LAD may create a more pro-atherogenic environment, particularly relevant in the absence of systemic risk enhancing factors. To the authors’ knowledge, this is the first study characterising atherosclerotic distributional differences in this population in a stable asymptomatic cohort with CACS measures.

Prior analyses of STEMI registries have consistently shown a higher frequency of LAD culprit lesions in SMuRFless participants, raising the possibility that these individuals may follow a different pattern of atherosclerotic involvement. Although anatomical, hemodynamic, and developmental factors have been proposed to explain this LAD susceptibility, the present observational analysis cannot evaluate these mechanisms. This may warrant further investigation, particularly with approaches capable of characterising non-calcified plaque and upstream vascular biology.

Embryologic and endothelial lineage differences have been proposed as potential contributors to vessel-specific differences in atherosclerotic development, including propensity of the LAD to develop early plaque. The initiation of the coronary network is known to occur through a common pathway beginning at approximately embryonic day 40.^27^ Increased myocardial thickening, metabolic demand, and hypoxia leads to the formation of a vascular plexus at the aortic root, ingrowth into the aortic wall, formation of the coronary ostium, and expansion into the coronary tree.^27^ The developmental programming of this complex process, involving proliferation and migration of numerous progenitor cells, remains incompletely understood. However, given its role in atherosclerosis development, territory-specific differences in coronary artery endothelial development have been identified. Lineage tracing studies have identified most coronary artery endothelial cells arising from angiogenesis originating from the sinus venosus or endocardium, with the latter primarily associated with septal and ventral cardiac angiogenesis.^28,29^ The historic reports of higher proportion of LAD culprit lesions in SMuRFless STEMI presentations, identified higher proportion of LAD CACS in SMuRFless MESA participants with a positive CACS at baseline in the present study, and theoretical opportunity for divergent developmental LAD biology specifically, together provides further justification to include people with CAD in the absence of SMuRFs as a group of special research interest.

Despite these novel findings, several limitations exist which warrant future research. Firstly, this analysis utilised non-contrast CT for CACS estimation. Differences in non-calcified plaque in SMuRFless participants, upstream to the development of coronary calcium, were unable to be explored. However, use of non-contrast cardiac CT remains a well-validated and widely accepted tool to risk stratify patients,^30,31^ with correlation to total atherosclerotic burden.^32,33^ In addition, CACS is known to be impacted by the initiation of statins in individuals with non-calcified atherosclerosis and can be a sign of plaque stabilisation. In this cohort, 18.8 % of the SMuRFless and 31.7 % of the SMuRF≥1 participants with a positive baseline CACS initiated statin therapy during follow-up, potentially confounding analyses of calcium score progression (Supplemental Table 7). However, a final model including statin initiation in this population was non-significant and did not alter the findings. Each SMuRF was classified using a binary definition, based on self-reported prior diagnosis or reaching a biomarker threshold used in primary prevention at baseline. However, these risk factors have demonstrated synergistic activity, confer risk on a continuous basis, and can have differential host responses between race and ethnicity groups. Sensitivity analyses using more stringent definitions (e.g., SBP <120/80 mmHg, LCL-C <100 mg/dL, glucose <100 mg/dL) could further explore this limitation, or those who develop a risk factor during follow-up, but were unable to be explored due to sample size. In general, our focus was to isolate individuals who develop coronary atherosclerosis despite an absence of SMuRFs, wherein heightened vascular susceptibility, rather than unmeasured lifestyle factors, may be most relevant. Although lifestyle and biomarker profiles, including lipoprotein (a), may refine future phenotyping and risk stratification, such analyses lie beyond the scope of the present study.

Finally, there are limitations with secondary analysis of observational cohort studies as a study design, which deserve consideration in the interpretations of results. Firstly, the MESA study was a prospective, observational cohort study, limiting the ability to interpret any causality of SMuRFless status on difference in LAD calcification. In addition, though the presence of hypertension, hypercholesterolaemia, diabetes mellitus, and smoking were removed in the SMuRFless individuals, there may be known and unknown CAD risk factors and exposures present in both groups, which could confound the results in the SMuRFless group. Classification of SMuRF status using self-report, biomarker, and/or treatment information, where available, minimises the opportunity for information bias; however, the possibility for this still exists.

## 5. Conclusion

SMuRFless participants of the MESA study with evidence of coronary calcification had a higher proportion of their coronary calcium in the LAD, compared to participants with at least one risk factor. Furthermore, these individuals experienced significantly greater progression of LAD calcification longitudinally. Together with prior findings of increased LAD culprit lesions in SMuRFless STEMI patients, our results underscore the LAD as a biologically relevant focal point of vulnerability in the SMuRFless population. These results justify further study, including into differences in non-calcified plaque, vascular biology, and preventative strategies for this increasingly recognised patient population.

## Supplementary Material

MMC2

MMC1

## Figures and Tables

**Table 1:** Baseline characteristics by SMuRF status.

Characteristic	Total Cohort N = 6792 Med (IQR) or No. %)	SMuRF = 0 N = 1397 (20.6 %) Med (IQR) or No. %)	SMuRF≥1 N = 5395 (79.4 %) Med (IQR) or No. %)	*P*

Age	62 (53, 70)	57 (50, 66)	64 (55, 71)	<0.0001
Male	3203 (47.2)	689 (49.3)	2514 (46.6)	0.069
Race/Ethnicity				
White	2615 (38.5)	588 (42.1)	2027 (37.6)	<0.001
Chinese	803 (11.8)	238 (17.0)	565 (10.5)	
Black	1878 (27.7)	266 (19.0)	1612 (29.9)	
Hispanic/Latino	1496 (22.0)	305 (21.8)	1191 (22.1)	
SBP	123.5 (111, 140)	112.5 (103.5, 122.5)	128 (114, 143.5)	<0.0001
DBP	71.5 (65, 78.5)	68.5 (62, 74.5)	72.5 (65.5, 79.5)	<0.0001
Body mass index (kg/m^2^)	27.6 (24.6, 31.2)	26.1 (23.3, 29.7)	27.9 (24.9, 31.6)	<0.0001
Total cholesterol (mg/dL)	192 (170, 215)	179 (162, 192)	198 (173, 222)	<0.0001
LDL cholesterol (mg/dL)	116 (96, 136)	105 (90, 118)	120 (99, 142)	<0.0001
HDL cholesterol (mg/dL)	48 (40, 59)	50 (41, 61)	48 (40, 58)	0.0002
Triglycerides	111 (78, 161)	92 (65, 132)	117 (83, 169)	<0.0001
Hypertension	3252 (47.9)	0 (0.0)	3252 (60.3)	n/a
Hypercholesterolaemia	3622 (53.3)	0 (0.0)	3622 (67.1)	n/a
Diabetes	865 (12.7)	0 (0.0)	865 (16.0)	n/a
Smoking	968 (14.3)	0 (0.0)	968 (17.9)	n/a
Fibrinogen	338 (295, 389)	317 (278, 363)	345 (300, 394)	<0.0001
CRP	1.92 (0.84, 4.25)	1.32 (0.60, 3.44)	2.08 (0.93, 4.43)	<0.0001
Creatinine	0.92 (0.82, 1.12)	0.92 (0.82, 1.02)	0.92 (0.82, 1.12)	<0.0001
10-year ASCVD risk				
Low (<5.0 %)	2158 (31.9)	831 (58.5)	1327 (24.8)	<0.001
Borderline (≥5.0 %, <7.5 %)	768 (11.4)	153 (11.0)	615 (11.5)	
Intermediate (≥7.5 %, <20.0 %)	2218 (32.8)	306 (21.9)	1912 (35.7)	
High (≥20.0)	1613 (23.9)	107 (7.7)	1506 (28.1)	
Annual gross income per household member ($10k)	2.08 (1.04, 3.33)	2.25 (1.13, 3.75)	2.08 (1.00, 3.25)	0.0003
Secondary education or higher	5566 (82.0)	1208 (86.5)	4358 (80.8)	<0.001
CACS>0 AU	3368 (50.0)	477 (34.4)	2891 (54.0)	<0.001
Total CACS, Agatston units (AU)	0.78 (0.0, 90.7)	0.0 (0.0, 20.3)	5.1 (0.0, 118.2)	<0.0001

**Table 2:** Bivariable and multi-variable analysis of baseline ln(LAD CACS + 1) (n = 6792).

Characteristic	Bivariable model^a^			Best-subsets model		
	
	Beta Coefficient	95 % CI	P	Beta Coefficient	95 % CI	P

SMuRF = 0	0.0474	0.00447, 0.0903	0.030			
Age	0.000338	−0.00155, 0.00222	0.73	0.000466	−0.00144, 0.00238	0.633
Male	0.000960	−0.0343, 0.0362	0.96	−0.00916	−0.0457, 0.0274	0.623
Race/Ethnicity						
White	Ref		<0.0001	Ref		
Chinese	0.000148	−0.0568, 0.0571		−0.0140	−0.0720, 0.0439	0.635
Black	−0.0378	−0.0807, 0.00508		−0.0296	−0.0731, 0.0140	0.183
Hispanic/Latino	−0.0904	−0.136, −0.0445		−0.0693	−0.111, −0.0276	0.001
Body mass index (kg/m^2^)	−0.00524	−0.00836, −0.00212	0.001			
Fibrinogen	−0.000383	−0.000617, −0.00150	0.001			
CRP (Log)	−0.0287	−0.0434, −0.0139	<0.001	−0.0262	−0.0418, −0.0105	0.001
Creatinine	−0.0279	−0.0894, 0.0336	0.374			
Annual gross income per household member ($k)	0.00554	−0.00351, 0.0146	0.230			
Secondary education or higher	0.0465	0.00204, 0.0910	0.040			
Total CACS, Agatston units (AU) [LN (+1)]	0.842	0.835, 0.849	<0.001	0.840	0.832, 0.848	<0.001

a Model adjusted for total coronary artery calcium score (CACS).

**Table 3:** Annualised increase in left anterior descending (LAD) coronary artery calcium score (CACS) progression in participants with a positive baseline CACS (n = 2925), by clinically relevant CACS strata and sex.

CACS Stratum Agatston Units	SMuRF Status	Total Cohort LAD CACS Progression (AU/year) Med (IQR)	Male LAD CACS Progression (AU/year) Med (IQR)	Female LAD CACS Progression (AU/year) Med (IQR)

1–99	SMuRF = 0	4.9 (0.8, 11.1)	4.6 (1.0, 10.7)	5.2 (0.6, 11.5)
	SMuRF≥1	4.4 (0.5, 9.9)	5.0 (0.9, 9.9)	2.9 (0.0, 9.9)
100–400	SMuRF = 0	14.3 (3.7, 29.0)	14.9 (4.2, 30.6)	13.2 (2.7, 28.1)
	SMuRF≥1	11.5 (2.6, 24.0)	11.6 (3.5, 21.3)	8.4 (0.0, 25.6)
401+	SMuRF = 0	20.6 (0.0, 52.5)	20.7 (0.0, 53.8)	20.5 (1.6, 51.0)
	SMuRF≥1	18.4 (1.3, 47.0)	20.1 (0.0, 47.0)	11.8 (1.4, 63.6)

**Table 4:** Bivariable and multivariable analysis of annualised ln(Change in LAD CACS + 1) at final scan in participants with a positive baseline CACS (n = 2382).

Characteristic	Multivariable model		
	Beta Coefficient	95 % CI	P

SMuRF = 0	0.0856	−0.0308, 0.202	0.149
Age	−0.00281	−0.00715, 0.00154	0.205
Male	−0.00379	−0.0880, 0.0804	0.930
Race/Ethnicity			
White	Ref		
Chinese	−0.152	−0.283, −0.0204	0.024
Black	−0.112	−0.216, −0.00718	0.036
Hispanic/Latino	−0.189	−0.298, −0.0799	<0.001
Total change in CACS from baseline, Agatston units (AU) [LN (+1)]	0.780	0.752, 0.809	<0.001

## Appendix A. Supplementary data

Supplementary data to this article can be found online at https://doi.org/10.1016/j.jcct.2026.01.004.

## Abbreviations:

AU: Agatston units
CACS: coronary artery calcium score
CAD: coronary artery disease
CCTA: coronary CT angiography
CVD: cardiovascular disease
DBP: diastolic blood pressure
LAD: left anterior descending
MESA: Multi-Ethnic Study of Atherosclerosis
SBP: systolic blood pressure
SD: standard deviation
SMuRF: Standard Modifiable cardiovascular Risk Factor
STEMI: ST elevation myocardial infarction
