# Supplementary material for: Disproportionate atherosclerotic burden in the left anterior descending coronary artery in participants without standard modifiable cardiovascular risk factors: The multi-ethnic study of atherosclerosis (MESA)
Source: J Cardiovasc Comput Tomogr. Author manuscript; Available in PMC 2026 May 9. (PMC13157333; doi:10.1016/j.jcct.2026.01.004)
Supplement: MMC2 [file NIHMS2167359-supplement-MMC2.docx]

Flow diagram to select eligible participants for analysis

Total MESA cohort at baseline examination in 2000-2002 (n=6,814)

(n = …)

# MESA Enrollment

Excluded (n = 22 [0.3%])

Missing smoking data

(n = 22)

Analysis Cohort (n = 6,792)

Analysis Cohort

#

Baseline CACS = 0 AU

(n = 3,446 [50.7%])

Baseline CACS >0 AU

(n = 3,368 [49.6%])
