# Supplementary material for: Disproportionate atherosclerotic burden in the left anterior descending coronary artery in participants without standard modifiable cardiovascular risk factors: The multi-ethnic study of atherosclerosis (MESA)
Source: J Cardiovasc Comput Tomogr. Author manuscript; Available in PMC 2026 May 9. (PMC13157333; doi:10.1016/j.jcct.2026.01.004)
Supplement: MMC1 [file NIHMS2167359-supplement-MMC1.docx]

Supplementary Materials

# Figure S1

Description: **Flow diagram to select eligible participants for analysis cohort.**


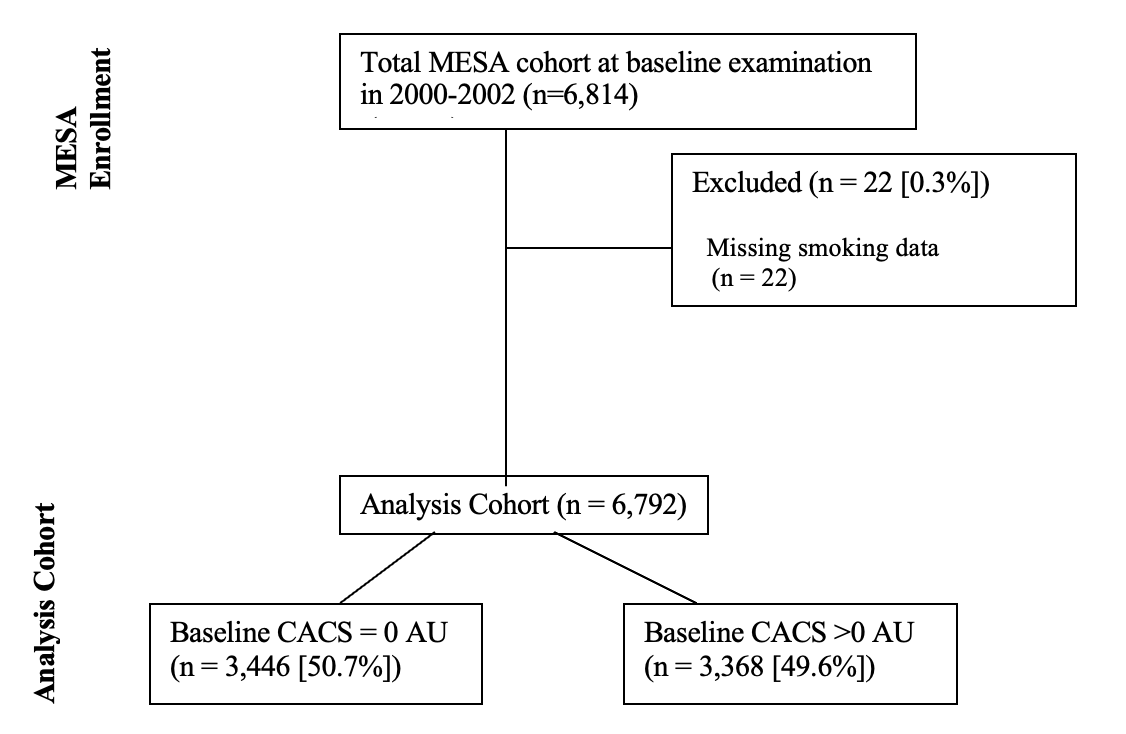


# Table S1

Description: **Baseline characteristics and coronary artery calcium data in participants with a positive coronary artery calcium score (CACS).**

| Characteristic | Total Cohort  N=3,368 (50.0%)  Med (IQR) or No. (%) | SMuRF=0  N=477 (14.2%)  Med (IQR) or No. (%) | SMuRF≥1  N=2,891 (85.8%)  Med (IQR) or No. (%) | *P* |
| --- | --- | --- | --- | --- |
| Age | 67 (59, 74) | 66 (57, 73) | 67 (60, 74) | 0.0011 |
| Male | 1,952 (58.0) | 322 (67.5) | 1,630 (56.4) | <0.001 |
| Race/Ethnicity  White  Chinese  Black  Hispanic/Latino | 1,476 (43.8)  408 (12.1)  811 (24.1)  673 (20.0) | 239 (50.1)  79 (16.6)  70 (14.7)  89 (18.7) | 1,237 (42.8)  329 (11.4)  741 (25.6)  584 (20.2) | <0.001 |
| SBP | 129.5 (115.5, 143.5) | 117.5 (108.5, 127) | 132.5 (117, 146.5) | <0.0001 |
| DBP | 72.5 (65.5, 79) | 69.5 (63.5, 75.5) | 72.5 (66, 80) | <0.0001 |
| Body mass index (kg/m^2^) | 27.7 (24.7, 31.1) | 26.5 (23.5, 29.8) | 27.9 (24.9, 31.3) | <0.0001 |
| Total cholesterol (mg/dL) | 192 (171, 216) | 180 (164, 192) | 196 (172, 220) | <0.0001 |
| LDL cholesterol (mg/dL) | 117 (96, 137) | 108 (93, 120) | 119 (98, 141.5) | <0.0001 |
| HDL cholesterol (mg/dL) | 47 (39, 57) | 46 (39, 58) | 47 (39, 57) | 0.8742 |
| Triglycerides | 117 (82, 116) | 96 (69, 137) | 121 (85, 172) | <0.0001 |
| Hypertension | 1,934 (57.4) | 0 (0.0) | 1,934 (100.0) | N/A |
| Hypercholesterolaemia | 1,983 (58.9) | 0 (0.0) | 1,983 (68.6) | N/A |
| Diabetes | 532 (15.8) | 0 (0.0) | 532 (18.4) | N/A |
| Smoking | 479 (14.2) | 0 (0.0) | 479 (16.6) | N/A |
| Fibrinogen | 345 (301, 394) | 320 (283.5, 368) | 349 (305, 399) | <0.0001 |
| CRP (Log) | 0.647 (-0.163, 1.428) | 0.239 (-0.511, 1.151) | 0.688 (-0.105, 1.452) | <0.0001 |
| Creatinine | 1.02 (0.82, 1.12) | 1.02 (0.82, 1.12) | 1.02 (0.82, 1.12) | 0.2912 |
| 10-year ASCVD risk  Low (<5.0%)  Borderline (≥5.0%, <7.5%) Intermediate (≥7.5%, <20.0%)  High (≥20.0) | 466 (13.9)  350 (10.4)  1,288 (38.4)  1,247 (37.2) | 141 (29.6)  68 (14.3)  184 (38.6)  84 (17.6) | 325 (11.3)  282 (9.8)  1,104 (38.4)  1,163 (40.5) | <0.001 |
| Annual Gross Income per Household Member ($k) | 21.9 (10.4, 33.3) | 22.5 (11.3, 43.8) | 20.8 (10.0, 32.5) | 0.0002 |
| Secondary Education or Higher | 2,739 (81.4) | 409 (85.9) | 2,330 (80.6) | 0.006 |
| Total CACS, Agatston units (AU) | 92.9 (24.5, 316.0) | 57.6 (17.1, 190.2) | 102.2 (25.7, 346.4) | <0.0001 |

# Table S2

Description: **Bivariable and multivariable analysis of ln(LAD CAC Volume +1) at baseline (n=6,792).**

| **Characteristic** | **Bivariable model^[[1]](#footnote-1)^** | | | **Multivariable model** | | |
| --- | --- | --- | --- | --- | --- | --- |
|  | Beta Coefficient | 95% CI | P | Beta Coefficient | 95% CI | P |
| SMuRF=0 | 0.0385 | -0.00424, 0.0813 | 0.077 |  |  |  |
| Age | 0.00106 | -0.000810, 0.00294 | 0.266 |  |  |  |
| Male | -0.00198 | -0.0371, 0.0332 | 0.912 |  |  |  |
| Race/Ethnicity  White  Chinese  Black  Hispanic/Latino | Ref  0.000798  -0.0349  -0.0906 | Ref  -0.0559, 0.0575  -0.0777, 0.00779  -0.136, -0.0449 | <0.0001 | -0.0701 | -0.0447, -0.0145 | <0.001 |
| Body mass index (kg/m^2^) | -0.00533 | -0.00844, -0.00222 | 0.001 |  |  |  |
| Fibrinogen | -0.000375 | -0.000608, -0.000143 | 0.002 |  |  |  |
| CRP (Log) | -0.0301 | -0.0448, -0.0154 | <0.001 | -0.0296 | -0.0447, -0.0145 | <0.001 |
| Creatinine | -0.0230 | -0.0842, 0.0382 | 0.462 |  |  |  |
| Annual Gross Income per Household Member ($k) | 6.3E^-7^ | -2.7E^-7^, 1.53E^-6^ | 0.170 |  |  |  |
| Secondary Education or Higher | 0.0416 | -0.00274, 0.085 | 0.066 |  |  |  |
| Total CAC Volume (AU) [LN(+1)] | 0.834 | 0.827, 0.841 | <0.001 | 0.833 | 0.826, 0.840 | <0.001 |

# Table S3

Description: **Bivariable and multivariable analysis of ln(LCx CACS +1) at baseline (n=6,792).**

| **Characteristic** | **Bivariable model^[[2]](#footnote-2)^** | | | **Multivariable model** | | |
| --- | --- | --- | --- | --- | --- | --- |
|  | Beta Coefficient | 95% CI | P | Beta Coefficient | 95% CI | P |
| SMuRF=0 | -0.0788 | -0.147, -0.0105 | 0.024 |  |  |  |
| Age | -0.00136 | -0.00436, 0.00164 | 0.373 |  |  |  |
| Male | 0.00500 | -0.0511, 0.0612 | 0.861 |  |  |  |
| Race/Ethnicity  White  Chinese  Black  Hispanic/Latino | -0.0230  -0.255  0.102  0.0681 | -0.0795, 0.0334  -0.339, -0.171  0.041, 0.163  0.00229, 0.134 | <0.0001 | -0.256 | -0.340, -0.171 | <0.001 |
| Body mass index (kg/m^2^) | 0.00995 | 0.00498, 0.0149 | <0.001 |  |  |  |
| CRP (Log) | 0.0162 | -0.00732, 0.0396 | 0.177 |  |  |  |
| Creatinine | 0.0505 | -0.0472, 0.148 | 0.311 |  |  |  |
| Annual Gross Income per Household Member ($k) | 9.9E^-7^ | -4.5E^-7^, 2.4E^-6^ | 0.177 |  |  |  |
| Secondary Education or Higher | -0.00612 | -0.0769, 0.0467 | 0.866 |  |  |  |
| Total CACS, Agatston units (AU) [LN(+1)] | 0.581 | 0.570, 0.591 | <0.0001 | 0.576 | 0.565, 0.587 | <0.001 |

# Table S4

Description: **Bivariable and multivariable analysis of ln(RCA CACS +1) at baseline (n=6,792).**

| **Characteristic** | **Bivariable model^[[3]](#footnote-3)^** | | | **Multivariable model** | | |  |
| --- | --- | --- | --- | --- | --- | --- | --- |
|  | Beta Coefficient | 95% CI | P | Beta Coefficient | 95% CI | P |  |
| SMuRF=0 | -0.0415 | -0.116, 0.0334 | 0.277 |  |  |  |  |
| Age | -0.00149 | -0.00478, 0.00179 | 0.373 |  |  |  |  |
| Male | 0.0976 | 0.0361, 0.159 | 0.002 | 0.0976 | 0.0361, 0.159 | 0.002 |  |
| Race/Ethnicity  White  Chinese  Black  Hispanic/Latino | -0.00760  -0.0603  -0.0213  0.0718 | -0.0694, 0.0542  -0.153, -0.0321  -0.0882, 0.0456  -0.000306, 0.144 | <0.0001 |  |  |  |  |
| Body mass index (kg/m^2^) | 0.00237 | -0.00308, 0.00782 | 0.394 |  |  |  |  |
| Fibrinogen | 0.0000872 | -0.00320, 0.000494 | 0.674 |  |  |  |  |
| CRP (Log) | 0.0117 | -0.0141, 0.0374 | 0.374 |  |  |  |  |
| Creatinine | 0.0213 | -0.0859, 0.128 | 0.697 |  |  |  |  |
| Annual Gross Income per Household Member ($k) | -4.3E^-7^ | -2.0E^-6^, 1.1E^-6^ | 0.588 |  |  |  |  |
| Secondary Education or Higher | 0.0442 | -0.0334, 0.121 | 0.265 |  |  |  |  |
| Total CACS, Agatston units (AU) [LN(+1)] | 0.569 | 0.557, 0.581 | <0.001 | 0.565 | 0.553, 0.577 | <0.001 |  |

# Table S5

Description: **Follow-up summary statistics of participants with a positive baseline CACS (n=2,925).**

| Characteristic | Total Cohort  N=2,925  Med (IQR) or No. (%) | SMuRF=0  N=426 (14.6%)  Med (IQR) or No. (%) | SMuRF≥1  N=2,499 (85.4%)  Med (IQR) or No. (%) | *P* |
| --- | --- | --- | --- | --- |
| Follow-up, Years | 8.8 (3.0, 9.5) | 8.9 (3.0, 9.5) | 8.7 (3.0, 9.5) | 0.4021 |
| CACS Progression | 2,681 (91.7) | 381 (89.4) | 2,300 (92.0) | 0.073 |
| Total change in CACS  Total CACS  LM  LAD  LCx  RCA | 110.75 (32.5, 311.6)  0.0 (0.0, 26.7)  41.1 (4.3, 118.9)  3.9 (0.0, 52.9)  9.8 (0.0, 82.2) | 74.3 (15.9, 218.3)  0.0 (0.0, 3.7)  36.5 (2.8, 93.1)  0.0 (0.0, 18.9)  1.1 (0.0, 44.7) | 117.8 (34.8, 338.8)  0.0 (0.0, 31.3)  41.8 (4.3, 124.3)  5.6 (0.0, 60.1)  11.9 (0.0, 87.8) | <0.0001  <0.0001  0.0404  <0.0001  <0.0001 |
| Annualised change in CACS, AU/year  Total CACS  LM  LAD  LCx  RCA | 20.8 (6.9, 54.1)  0.0 (0.0, 4.5)  7.4 (0.9, 20.3)  0.7 (0.0, 9.5)  1.8 (0.0, 14.6) | 12.7 (4.7, 32.7)  0.0 (0.0, 0.8)  6.0 (0.7, 14.3)  0.0 (0.0, 4.0)  0.3 (0.0, 7.5) | 23.1 (7.6, 57.6)  0.0 (0.0, 5.0)  7.7 (1.0, 21.2)  1.0 (0.0, 10.7)  2.3 (0.0, 15.9) | <0.0001  <0.0001  0.0081  <0.0001  <0.0001 |

#

# Table S6

Description: **Summary of statin therapy at baseline and changes in follow-up for all participants, by SMuRF status (n=6,792).**

| Baseline SMuRF Status | Baseline Statin  No. (%) | Statin Maintained  No. (%) | Statin Initiated  No. (%) | Statin Discontinued  No. (%) |
| --- | --- | --- | --- | --- |
| SMuRF = 0  (n=1,397, 20.6%) | 0 (0.0%) | 0 (0.0%) | 169 (12.1%) | 0 (0.0%) |
| SMuRF ≥ 1  (n=5,395, 79.4%) | 1,100 (20.4%) | 997 (18.5%) | 1,643 (30.5%) | 0 (0.0%) |

# Table S7

Description: **Summary of statin therapy at baseline and changes in follow-up for participants with a positive baseline CACS, by SMuRF status (n=3,412).**

| Baseline SMuRF Status | Baseline Statin  No. (%) | Statin Maintained  No. (%) | Statin Initiated  No. (%) | Statin Discontinued  No. (%) |
| --- | --- | --- | --- | --- |
| SMuRF = 0  (n=489, 14.3%) | 0 (0.0%) | 0 (0.0%) | 92 (18.8%) | 0 (0.0%) |
| SMuRF ≥ 1  (n=2,923, 85.7%) | 677 (23.2%) | 677 (23.2%) | 927 (31.7%) | 0 (0.0%) |

1. Model adjusted for total coronary artery calcium score (CACS) [↑](#footnote-ref-1)
2. Model adjusted for total coronary artery calcium score (CACS) [↑](#footnote-ref-2)
3. Model adjusted for total coronary artery calcium score (CACS) [↑](#footnote-ref-3)
